# The double-edged sword: COVID-19 pandemic-related delay in immune maturation in young children

**DOI:** 10.1016/j.clinsp.2023.100239

**Published:** 2023-06-16

**Authors:** Braian Lucas Aguiar Sousa, Magda Maria Sales Carneiro-Sampaio

**Affiliations:** Faculdade de Medicina da Universidade de São Paulo (FMUSP), São Paulo, SP, Brazil

In 2020 the world was taken by surprise with the rise of a new and potentially fatal respiratory disease: COVID-19. As the disease rapidly spread worldwide, governments were forced to take severe restrictive measures and implement mask mandates to mitigate the impact of the virus and limit the death toll. In many ways, especially in 2020, these measures flattened the curve of cases,^1^ helping health systems to cope with demand and buying time for the science community to develop vaccines against the virus. However, COVID-19 was only one respiratory virus in a world that was already dominated by them, and the measures taken to cope with COVID-19 were bound to affect all viruses that spread in a similar fashion, deeply changing the dynamics of respiratory viral disease transmission.

Respiratory viral diseases are especially common among young children and are the leading cause of emergency department visits.^2^ Children, particularly those in pre-school years, are estimated to have up to 10 upper respiratory infections per year.^3^ Some might also develop lower respiratory infections, with more severe consequences. The dynamics of respiratory viral transmission usually follow a seasonal pattern, related to the common collective habits in each season. Usually, the transmission is higher in fall and winter, when people tend to spend more time indoors, facilitating disease transmission. Moreover, studies have shown that lower humidity and lower air temperatures might also affect host immunity against respiratory viruses, such as influenza.^4^^,^^5^ In Brazil, the seasonality of viral infections is influenced by latitude, being different among the regions. In the southwest, respiratory syncytial virus peaks from February to July, and influenza during the winter months.^6^^,^^7^ Fig. 1 shows the number of hospitalizations by respiratory infections in the studied country in children younger than 5 years, as a proxy of viral transmission. The year 2019 illustrates the usual behavior, with a peak of hospitalizations between March and June.

**Fig. 1:**
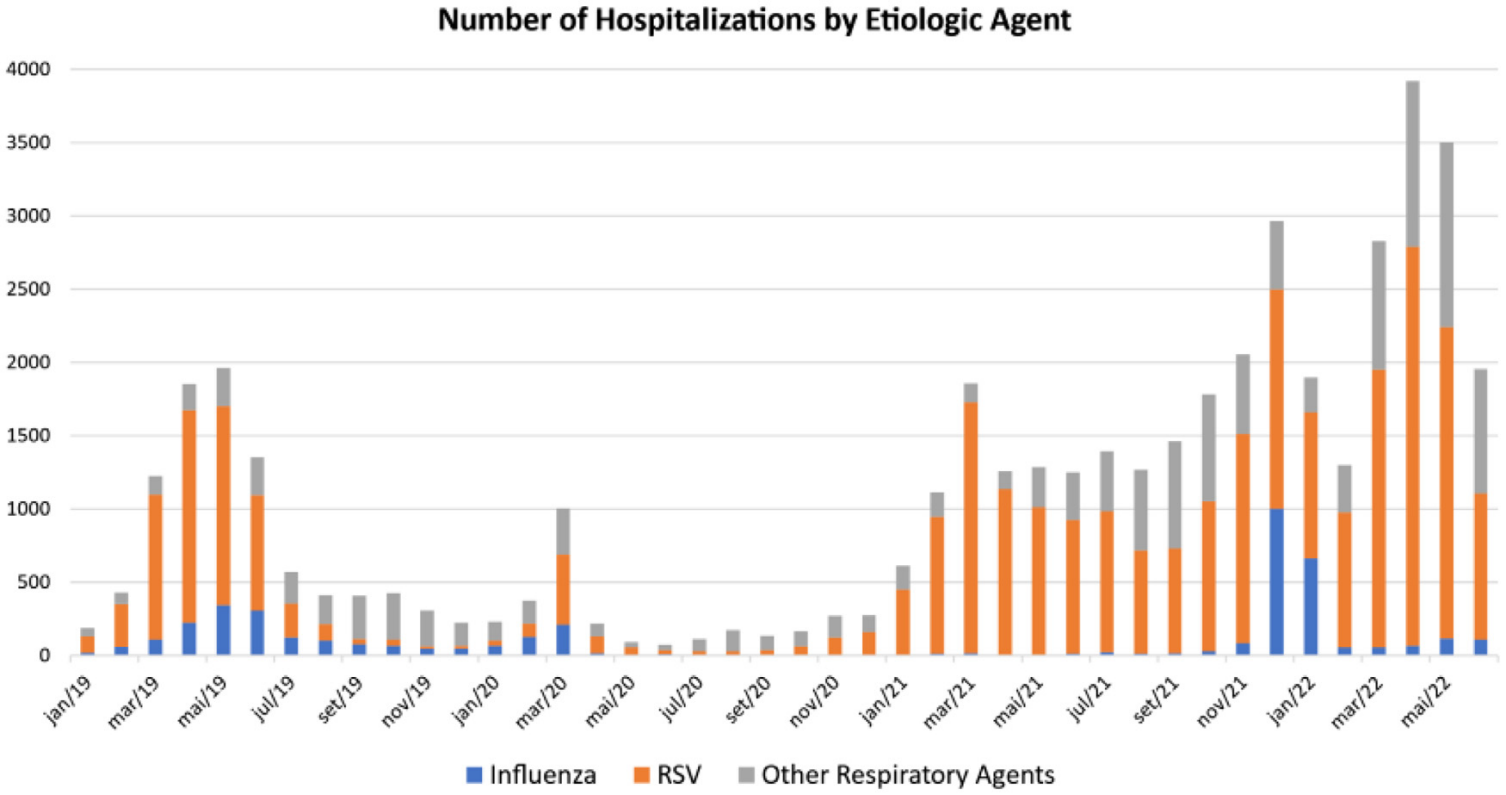
Number of severe acute respiratory syndrome hospitalizations by etiologic agent from January 2019 to June 2022. Data from the Sistema de Vigilância Epidemiológica da Gripe (SIVEP-Gripe) dataset. All patients younger than 5 years, hospitalized and with a respiratory virus isolated were included. Other Respiratory Agents: parainfluenza 1, parainfluenza 2, parainfluenza 3, parainfluenza 4, adenovirus, rhinovirus, bocavirus, metapneumovirus. RSV: Respiratory Syncytial Virus.

Many studies have shown disruption in respiratory viral seasonality during and after 2020.^8^^,^^9^ Due to isolation, masking mandates, reinforced hygiene measures, and school closure, the activity of most respiratory viral diseases other than COVID-19 sharply dropped in 2020, rising again after those measures were relaxed.^10^ In Brazil, the authors saw the same trend, as also illustrated by Fig. 1. COVID-19 hit the country in March when the seasonal peak was beginning to show its shape, but it was quickly suppressed by the social and behavioral changes that characterized the first months of the pandemic. By the end of the year, when those measures were relaxed, there was a peak in hospitalizations, that again was reduced by reinforced social measures, driven by the rising COVID-19 levels. However, it was in 2022 that respiratory infections reached their highest levels, leading to overcrowded hospitals and hours of waiting at pediatric emergency departments. The number of hospitalizations was even much higher than pre-pandemic levels, which led us to the question: has the pandemic delayed immune maturation among young children? The answer to this question invites us to reflect on how the immune system matures, how the pandemic might have affected this maturation, and to question what effects these children born during or a little before the pandemic might suffer in the long term.

Every pediatrician who cares for young children listens almost daily to parents complaining about recurrent viral infections. “He/she is always sick”, they ask, “is this normal? Isn't there a problem with his/her immunity?”. As a matter of fact, there is. And the problem is that, like most of his/her other organs and systems, the immune system is immature and must be “educated” over time about the threats that live in the outer world. This process is physiological and must be experienced by everyone, with some, especially those children who attend daycare, experiencing it faster (which can generate more anxiety among parents). The immunological mechanisms behind this immaturity are worth reviewing.

Firstly, concerning adaptive immunity, children, especially infants, do not have the same level of antibodies as adults. At the end of the first semester, we have low IgG, IgM, and IgA levels, that gradually increase over time, reaching adult levels around 6‒8 years for IgG and early adolescence for IgA. After these first 6 months, when the transplacental transfer of antibodies gives some protection, the child enters a physiological hypogammaglobulinemia phase.^11^ In addition, young children are not very efficient at producing anti-polysaccharide antibodies, which are important in the defense against encapsulated bacteria, such as *S. pneumoniae*, the most frequent agent of pneumonia.^12^

The maturity of antibody development depends on exposure to environmental antigens and the development of memory T and B cells. Children are characteristically born with low memory T and B cells, reflecting their naivety to the environment, and those levels progressively rise as they are exposed to new antigens and develop memory.^13^^,^^14^ Exposure to respiratory viruses and bacteria and the development of memory follow the same principles: it is necessary to have been exposed to the agents to “remember” them. Therefore, low exposure secondary to pandemic non-pharmaceutical interventions generates an “immunity debt” that must be eventually “paid”, as already pointed out by researchers.^15^^,^^16^ In addition, both the pandemic and the dissemination of fake news contributed to lower levels of vaccination (anti-COVID and others),^17^, ^18^, ^19^ thus increasing the debt.

Secondly, as the first line of defense against pathogens, innate immunity might also be involved. Cells and receptors that make the innate immunity are, by principle, unspecific ones. They defend us by quickly sensing patterns present in microbes and eliciting inflammation, directly destroying the foreign agent, and making the bridge to adaptive immunity. Innate immunity is responsible for eliminating most agents that infect us every day.^20^ Up until very recently, it was believed that only the adaptive immunity branch, represented by T and B lymphocytes, could mount memory, and protect against recurrent infections. However, new studies show that after exposure, innate immunity is also capable of displaying adaptive characteristics and developing memory, a feature that has been called “trained immunity”. The molecular mechanisms underlying these phenomena are epigenetic and lead to the upregulation of inflammatory gene expression, granting a faster and more efficient response after re-exposure.^21^^,^^22^ Trained immunity was hypothesized as one of the reasons children are less susceptible and manifest less severe cases of COVID-19, as they were more exposed to viral infections before the pandemic.^15^ On the other hand, the lack of “training” during the pandemic might have contributed to the explosion of respiratory viral infections in 2022.

The possible impact of social distancing and hygiene measures might extrapolate the infectious field. Concerns over how these measures might affect the development of allergies and autoimmunity were also raised,^15^ although definitive answers on these fields are still lacking. A birth cohort in Ireland including 365 infants born during the first COVID-19 lockdown (the CORAL cohort) is underway to address these questions and preliminary results already show a higher frequency of atopic dermatitis and higher rates of egg sensitization.^23^ Another study showed an increase in hand eczema among children secondary to the intensification of hand hygiene measures.^24^ Many of these consequences can be attributed at least partially to the role of microbiome diversity in the development of allergies and autoimmunity. As a concept derived from the hygiene theory, microbiome, and host interaction is currently among the most studied fields in immunology. There are many ways by which the pandemic might have disrupted microbiome homeostasis, including reduced social gatherings, increased use of antibiotics, and increased use of detergents, among others, leading to a shift from homeostasis to dysbiosis.^25^ The impact of the pandemic on microbiome homeostasis and the development of allergies and autoimmunity are also under investigation by the CORAL cohort. It is important to note that many studies have associated COVID-19 infections with the development of autoimmunity, especially among adults.^26^^,^^27^

The non-pharmacological measures adopted during the pandemic were, without doubt, essential to contain COVID-19 spreading and saving lives. However, they might have been a double-edged sword for young children, as delayed immune maturity might have increased susceptibility to infections and led to an explosion in respiratory viral diseases. Additionally, it is still early to evaluate the impact on allergy and autoimmunity development, and studies are underway. Pediatricians must be vigilant to these conditions, specifically among children born during the pandemic. Understanding the dynamics of respiratory viral infections, how it was disturbed by the COVID-19 pandemic and the possible future consequences of this phenomenon can better prepare us to face future challenges in community healthcare and provide better care for children.

## Conflicts of interest

The authors declare no conflicts of interest.
